# Novel function of LHFPL2 in female and male distal reproductive tract development

**DOI:** 10.1038/srep23037

**Published:** 2016-03-11

**Authors:** Fei Zhao, Jun Zhou, Rong Li, Elizabeth A. Dudley, Xiaoqin Ye

**Affiliations:** 1 Department of Physiology and Pharmacology, College of Veterinary Medicine, University of Georgia, Athens, GA 30602, USA; 2 Interdisciplinary Toxicology Program, University of Georgia, Athens, GA 30602, USA

## Abstract

Congenital reproductive tract anomalies could impair fertility. Female and male reproductive tracts are developed from Müllerian ducts and Wolffian ducts, respectively, involving initiation, elongation and differentiation. Genetic basis solely for distal reproductive tract development is largely unknown. *Lhfpl2* (lipoma HMGIC fusion partner-like 2) encodes a tetra-transmembrane protein with unknown functions. It is expressed in follicle cells of ovary and epithelial cells of reproductive tracts. A spontaneous point mutation of *Lhfpl2* (LHFPL2^G102E^) leads to infertility in 100% female mice, which have normal ovarian development, ovulation, uterine development, and uterine response to exogenous estrogen stimulation, but abnormal upper longitudinal vaginal septum and lower vaginal agenesis. Infertility is also observed in ~70% mutant males, which have normal mating behavior and sperm counts, but abnormal distal vas deferens convolution resulting in complete and incomplete blockage of reproductive tract in infertile and fertile males, respectively. On embryonic day 15.5, mutant Müllerian ducts and Wolffian ducts have elongated but their duct tips are enlarged and fail to merge with the urogenital sinus. These findings provide a novel function of LHFPL2 and a novel genetic basis for distal reproductive tract development; they also emphasize the importance of an additional merging phase for proper reproductive tract development.

The reproductive tract is essential for successful reproduction in mammals. The female reproductive tract, which includes oviduct, uterus, cervix, and vagina, coordinately provides suitable environments in different parts of the tract for mating, fertilization, embryo transport, embryo implantation, embryo development, and fetus delivery. The male reproductive tract, which consists of efferent duct, epididymis, vas deferens, and ejaculatory duct (union of vas deferens and duct of seminal vesicle), provides a lengthy channel for sperm maturation, sperm transport, sperm storage, and sperm delivery via ejaculation^1^. Congenital malformations of the female and male reproductive tracts can impair fertility^2^,^3^. Many genes (e.g., paired-box gene 2 (*Pax2*)) essential for reproductive tract development have been identified from genetically modified mouse models^2^,^4^,^5^,^6^. However, the genetic basis solely for distal reproductive tract development is largely unknown. Mouse models continue to be important for revealing novel genetic basis of reproductive tract development.

The female and male reproductive tracts are derived from a pair of paramesonephric ducts (or Müllerian ducts, MD) and a pair of mesonephric ducts (or Wolffian ducts, WD), respectively^4^. The reproductive tract development involves triphasic process of initiation, elongation and differentiation. Before sexual differentiation, the mammalian embryos are sexually indifferent with both MD and WD. In mice, WD initiates from the intermediate mesoderm at around embryonic day 9 (E9) and elongates craniocaudally^5^, while MD initiates from an invagination of mesonephros surface epithelium at around E11.5 and elongates following WD to reach the urogenital sinus by E13.5^4^,^7^,^8^. Subsequently, in female embryo, the WD degenerates and the MD differentiates into morphologically and functionally different subsections of the reproductive tract, i.e. oviduct, uterus, cervix, and upper vagina, which will extend to form the lower vagina during postnatal development^4^,^8^,^9^; while in male embryo, the MD degenerates and the WD differentiates into epididymis, vas deferens, seminal vesicle, and ejaculatory duct^4^.

LHFPL2 belongs to the lipoma HMGIC (high mobility group protein isoform I-C) fusion partner (LHFP) gene family, which includes six members, LHFP and LHFPL1-LHFPL5 that are predicted to be four transmembrane proteins with 200–247 amino acids in both human and mouse (NCBI). Since the identification of LHFP in 1999^10^, only a few papers have referred to LHFP gene family with LHFPL5 (or TMHS, tetraspan membrane protein of hair cell stereocilia) being the most studied member. Mutations in *Lhfpl5* could cause hearing disorders in humans^11^,^12^ and mice^13^,^14^,^15^,^16^,^17^, as well as hypospadias, anal atresia with a recto-urethral fistula, and hypoplastic kidney in a young boy^18^. Interestingly, a spontaneous mutation in *Lhfpl2* also causes hearing impairment in mice, in addition to corneal opacity and imperforate vagina (The Jackson Laboratory).

During the study of endocrine disruptors, we found that a few estrogenic endocrine disruptors could promote vaginal opening^19^,^20^,^21^,^22^,^23^, an early indicator of pubertal onset in mice^24^,^25^,^26^,^27^,^28^. It was our initial intention to use this *Lhfpl2* mutant as a loss-of-function model for studying mechanisms of vaginal opening, which are not well understood. However, our study led us away from the initial intention and to the novel findings that LHFPL2 is essential for distal reproductive tract development in both female and male mice.

## Results

### Lhfpl2 and Lhfpl2 point mutation

Since very limited information is available about the expression of *Lhfp* gene family in mice, RT-PCR (see Supplementary Fig. S1) was performed on several adult tissues, including heart, spleen, liver, kidney, thymus, testis, brain, lung, intestine, vagina, ovary and uterus. Among the six members of the *Lhfp* gene family, *Lhfp* and *Lhfpl2* were ubiquitously expressed in these tissues, *Lhfpl1* was detected in most tissues, *Lhfpl3*, *Lhfpl4*, and *Lhfpl5* had more tissue specific expression patterns with the highest expression in the brain. *Lhfpl2* was highly detected in the female reproductive system, such as ovary, uterus and vagina. The expression levels in ovary and uterus seemed comparable between 3 weeks old and 8 weeks old.

There are 7 mouse *Lhfpl2* transcripts indicated in Ensembl (http://www.ensembl.org/index.html). They encode proteins of 222aa (three transcripts), 139aa (one transcript), 34aa (one transcript), or no protein product (two transcripts). The full-length LHFPL2 protein (222aa) is predicted to be a tetra-transmembrane protein with a molecular weight of ~23.9 kD (see Supplementary Fig. S2a). It has both N and C termini in the cytoplasm, one intracellular loop between transmembrane domains 2 and 3, and two extracellular loops between transmembrane domains 1 and 2 as well as 3 and 4 (see Supplementary Fig. S2b). Based on the full-length *Lhfpl2* transcript (*Lhfpl2*-001), the *Lhfpl2* mutant mice (MUT/ LHFPL2^G102E^) used in this study have a spontaneous mutation in the 676^th^ nucleotide from G to A (see Supplementary Fig. S3a), resulting in a missense mutation of the 102^nd^ amino acid from nonpolar glycine (G) to negatively charged glutamic acid (E) (see Supplementary Fig. S3b) located on the predicated second transmembrane domain (see Supplementary Fig. S2c). This transition mutation creates a BglII restriction site (see Supplementary Fig. S3b) and can be confirmed by both restriction digestion coupled with PCR (see Supplementary Fig. S3c) and SNP genotyping (see Supplementary Fig. S3d).

The average litter size from heterozygous (HET) *Lhfpl2* females mated with HET males was 7.8 ± 2.3 (N = 16). The offspring from HET × HET crosses followed the Mendelian inheritance, with 21.8% WT, 52.8% HET, and 25.4% MUT at weaning. The LHFPL2^G102E^ mice generally appeared healthy except imperforate vagina in all females and corneal opacity and exophthalmos in both genders. The Jackson Laboratory also reported abnormal pupillary reflex, thin retinal inner nuclear layer, hearing problem, and abnormal perineum morphology in the MUT mice. Defects in male reproductive tract was identified later via functional analyses in our study. When LHFPL2^G102E^ mutant mice expressed BAC mouse *Lhfpl2* transgene, the female MUT/BAC+ mice had normal vagina and vaginal opening and the male MUT/BAC+ mice were fertile, confirming that this *Lhfpl2* point mutation was responsible for the phenotypes in both female and male reproductive tracts.

### Vaginal agenesis caused by defective distal MD development in Lhfpl2 mutant females

**Figure 1:**
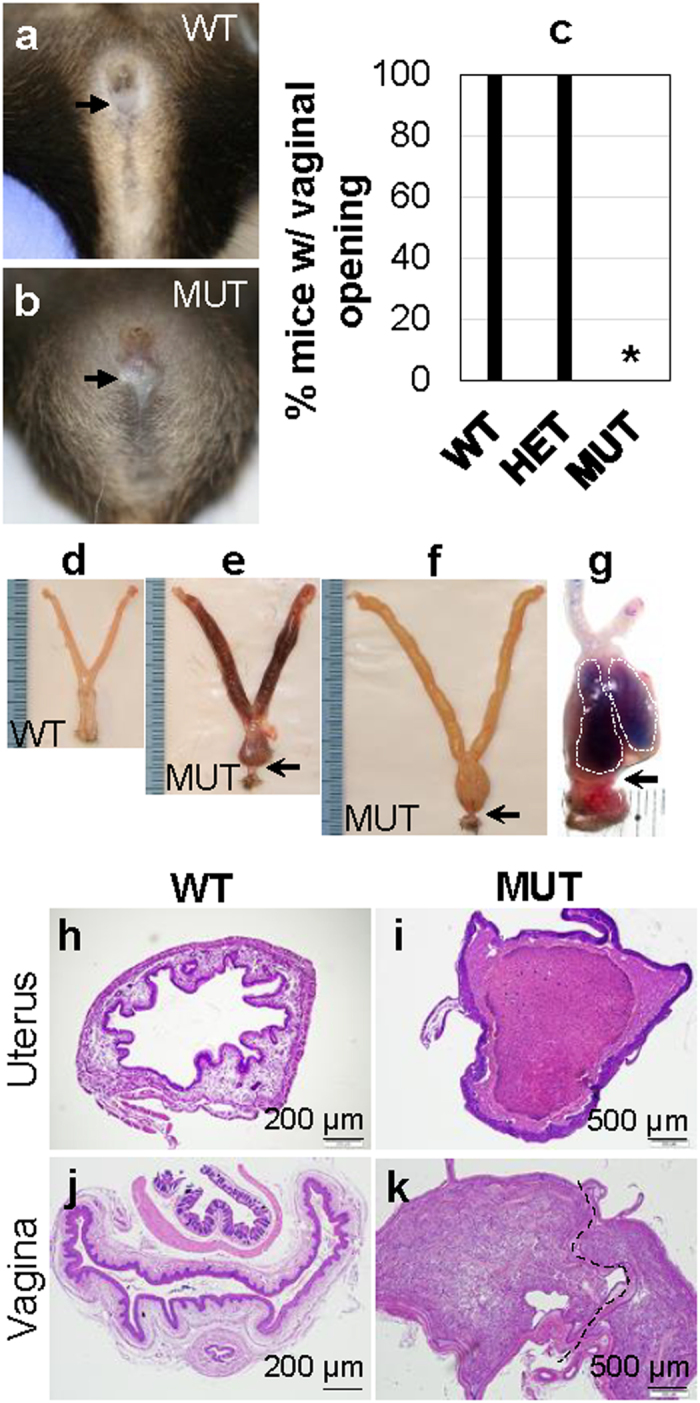
Imperforate vagina in *Lhfpl2* mutant (MUT) mice. (**a,b**) Representative pictures of WT (**a**) and MUT (**b**) mice at 8-week old. (**c**) Percentage of mice with vaginal opening. *P < 0.05. N = 18, 26 and 35 for WT, HET and MUT, respectively. (**d–f**) Representative reproductive tracts of WT (**d**) and MUT females (**e,f**) at 8-week old. (**g**) A MUT vagina showing two chambers (white dotted lines) by blue dye infusion. (**h**) Histology of a WT uterus. (**i**) Histology of a MUT uterus. (**j**) Histology of a WT vagina. (**k**) Histology of a MUT vagina. Dotted black line in (**k**), vaginal septum; black arrow, the position of vagina opening (**a,b**) or the position of vagina atresia (**e–g**); scale bar, 200 μm (**h**,**j**) or 500 μm (**i**,**k**).

Vaginal closure was observed in 100% of the MUT but none of WT or HET females at 8-week old (Fig. 1a–c). Vaginal closure prevented mating. Compared to the control (WT/HET) female reproductive tracts at the estrus stage when the uterus had the biggest size during estrous cycle (Fig. 1d), all the MUT female reproductive tracts were bigger with 30% of them having a dark appearance in the uterus and the upper vagina (Fig. 1e,f). Besides distended uterine horns and upper vagina, each MUT vagina lacked the lower vagina and had double lumens in upper vagina visualized by blue dye (Fig. 1e–g). Histology of 8-week old WT uterine horn and vagina at estrous stage showed a typical edematous stromal compartment and an open lumen in the uterus (Fig. 1h) as well as a single lumen vagina (Fig. 1j). Each MUT uterine horn had an enlarged lumen filled with a viscous fluid and had a compressed uterine wall (Fig. 1i). Each MUT upper vagina had enlarged double lumens filled with a viscous fluid and had a compressed vaginal wall (Fig. 1k), consistent with the double lumens indicated by blue dye infusion (Fig. 1g).

**Figure 2:**
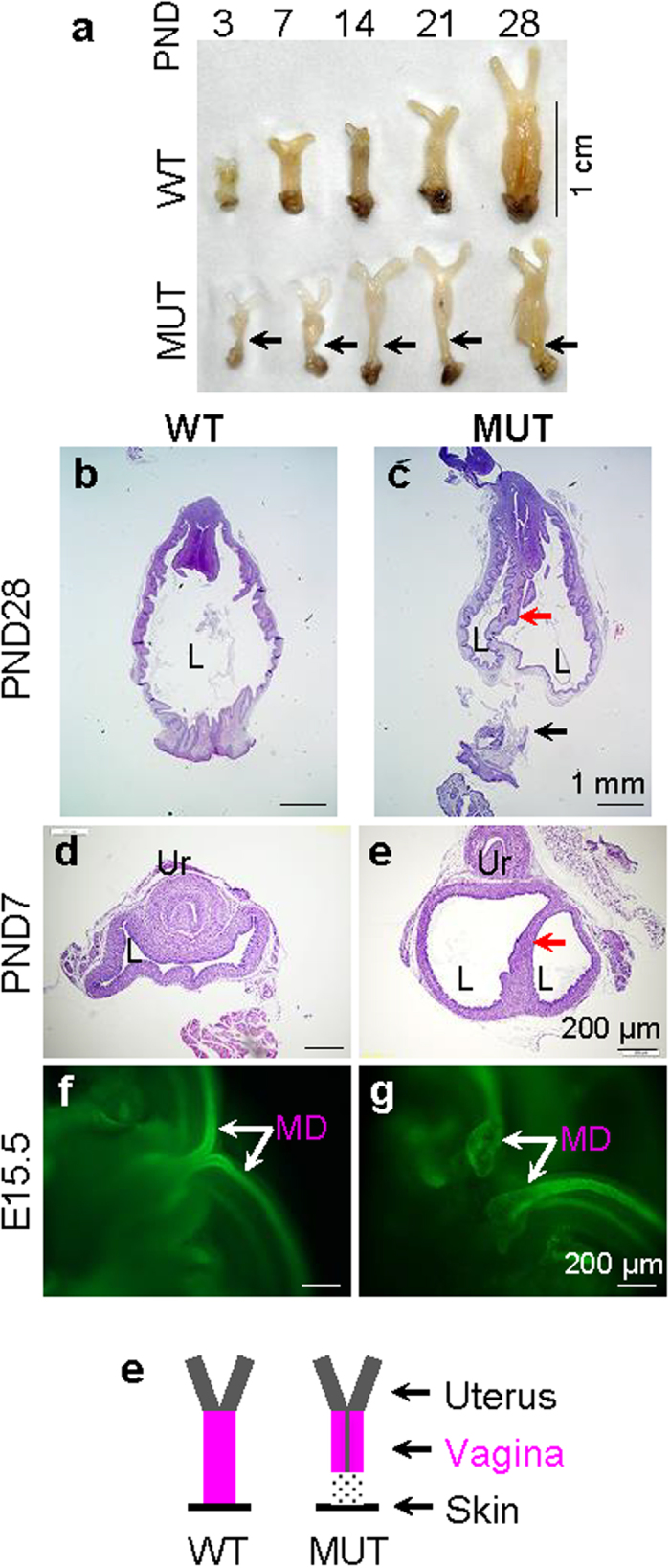
Vaginal development. (**a**) The appearance of WT and MUT vaginas at postnatal day (PND) 3, 7, 14, 21, and 28. (**b**) A longitudinal section of a WT vagina at PND28 showing single lumen. (**c**) A longitudinal section of a MUT vagina at PND28 showing double-lumen upper vagina and lower vagina atresia. (**d**) A cross section of a WT vagina at PND7. (**e**) A cross section of a MUT upper vagina at PND7 showing double-lumen. (**f**) PAX2 staining of a WT E15.5 female embryo showing Y-shaped Müllerian ducts. (**g**) PAX2 staining of a MUT E15.5 female embryo revealing unmerged and enlarged Müllerian duct tips. Black arrow, position of vaginal atresia; red arrow, medial wall/vaginal septum in MUT vagina; L, vaginal lumen; Ur, urethra; MD, Müllerian duct; scale bar, 1 cm (**a**), 1 mm (**b,c**), 200 μm (**d–g**). (**h**) Summary of the phenotypes in MUT female reproductive tract, including unfused upper vagina with medial wall and lower vaginal atresia. Grey line in MUT, vaginal septum; grey pattern in MUT, absence of lower vagina.

Analysis of postnatal/prepubertal vaginas showed distal vagina agenesis at all these ages examined from PND3 to PND28, so was double-lumen upper vagina in the MUT females (Fig. 2a–e). PAX2 staining of E15.5 female embryos revealed that the two WT MDs had partially fused to form a Y-shape and entered the urogenital sinus (Fig. 2f), while the two MUT MDs had enlarged tips that failed to merge with each other and failed to enter the urogenital sinus (Fig. 2g). The two MUT WDs also had enlarged tips that failed to enter the urogenital sinus that were not seen in the WT WDs (Fig. 2f,g). These data demonstrated that the MUT MDs had gone through initiation and elongation phases to reach the urogenital sinus, but they had defective tip development and failure of merging with urogenital sinus during embryonic stage, eventually resulting in unmerged upper vagina and lower vaginal agenesis (Fig. 2e).

### Expression of Lhfpl2 in the female reproductive system

**Figure 3:**
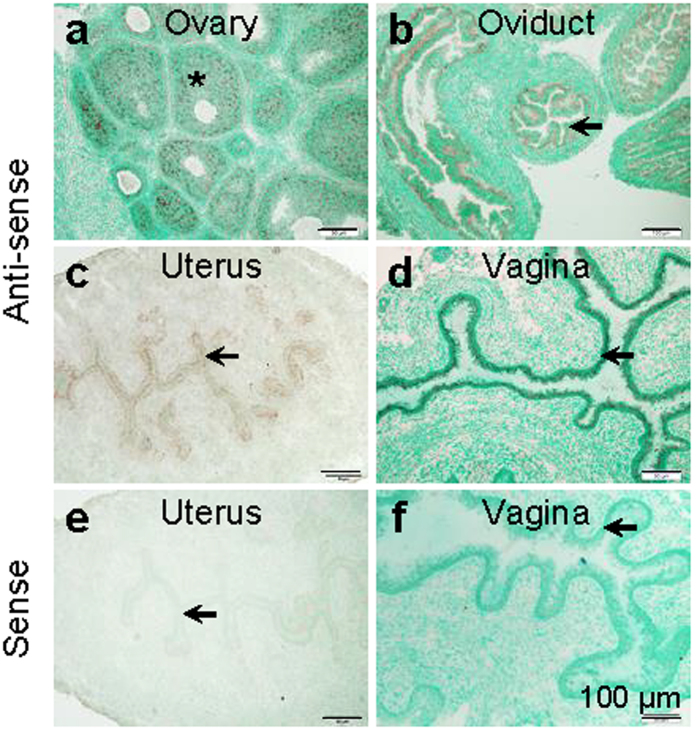
Localization of *Lhfpl2 *mRNA in 4 weeks old female reproductive tract by *in situ* hybridization. (**a**–**d)** Detection of *Lhfpl2 *mRNA using an *Lhfpl2* anti-sense probe in ovary (**a**), oviduct (**b**), uterus (**c**) and vagina (**d**). (**e**,**f** ) Negative control using an *Lhfpl2* sense probe in uterus (**e**) and vagina (**f** ). *Follicle; black arrow, epithelium; scale bar, 100 μm.

*In situ* hybridization of female reproductive system at 4 weeks old indicated that *Lhfpl2 *mRNA was mainly detected in follicle cells of the ovary (Fig. 3a), epithelial cells of the oviduct (Fig. 3b), both luminal and glandular epithelial cells of the uterus (Fig. 3c,e), and epithelial cells of the vagina (Fig. 3d,f).

### Normal ovarian function and uterine response to E2 in Lhfpl2 mutant females

**Figure 4:**
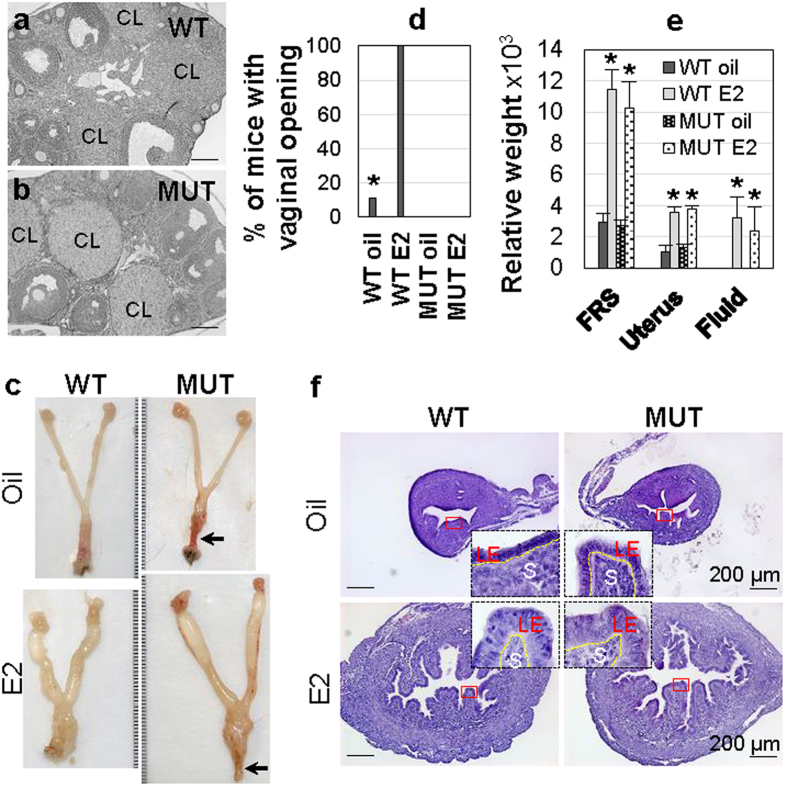
Ovary histology and uterine response to E2 treatment for 3 days. (**a**,**b**) Histology of 8-week old WT (**a**) and MUT (**b**) ovaries. CL, corpus luteum. (**c)** Representative images of 24- day old WT and MUT female reproductive system upon vehicle (oil) or E2 treatment. Black arrow, vaginal atresia. (**d)** Percentage of WT and MUT mice with vaginal opening upon oil or E2 treatment. (**e**) Relative weight of WT and MUT female reproductive system (FRS), uterus, and fluid upon oil or E2 treatment. Error bar, standard deviation; N = 5–9 (**c–e**); *P < 0.05 compared to vehicle control. (**f**) Representative WT and MUT uterine histology upon oil or E2 treatment. Insert in each panel, enlarged view of the red rectangle area; LE, uterine luminal epithelium; S, stroma; scale bar, 200 μm.

The high expression levels (see Supplementary Fig. S1) and cell type specific expression patterns of *Lhfpl2* in the ovary and the uterus (Fig. 3) promoted us to investigate potential roles of LHFPL2 in the ovary and the uterus. Although distal vaginal agenesis in 100% of *Lhfpl2* MUT females (Fig. 1) prevented investigation of ovarian and uterine functions through pregnancy, histology and E2 treatment provided important information about the functions of ovary and uterus in the MUT females. Histology showed that WT and MUT females had comparable ovary histology at 4-week old (data not shown) and 8-week old, when follicles at different stages and corpora lutea were present in both WT and MUT ovaries (Fig. 4a,b), indicating that the MUT ovary had normal development and ovulation. E2 treatment on newly weaned females (prior to vaginal opening and estrous cyclicity) revealed that E2 treatment accelerated vaginal opening in the WT females but the MUT vaginas remained closed (Fig. 4d). Both WT and MUT female reproductive tracts had comparable responses to E2 treatment, such as enlarged uteri and vaginas (Fig. 4c,e,f), fluid accumulation in the reproductive tracts (Fig. 4c,e), and taller uterine luminal epithelium (Fig. 4f), demonstrating that the MUT uterus had normal development and was functional in responding to E2 treatment. These data also confirmed that the MUT MDs had normal triphasic process of initiation, elongation, and differentiation.

### Impaired fertility in MUT males

**Figure 5:**
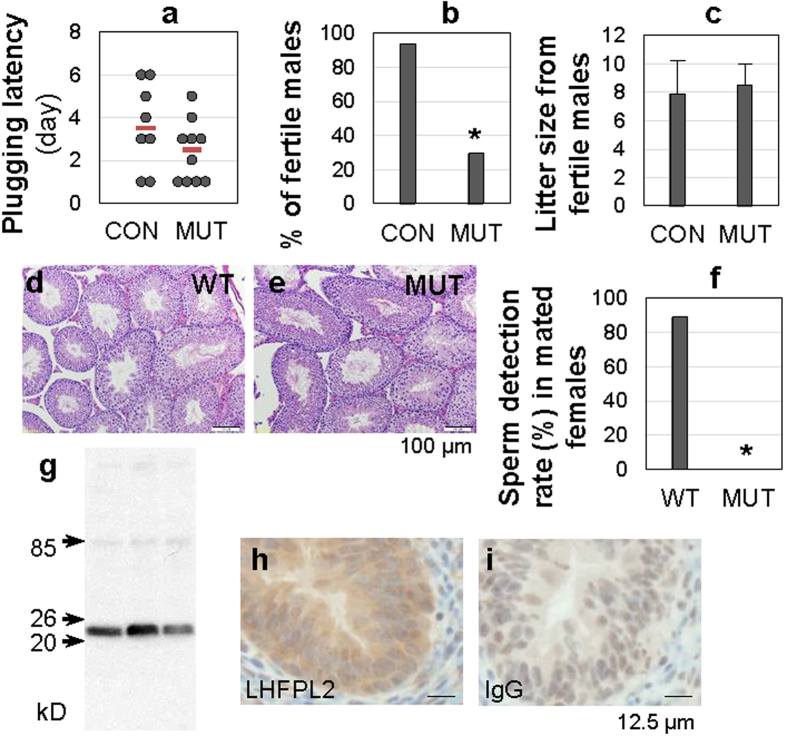
Male fertility test and localization of LHFPL2 in vas deferens. (**a**) Plugging latency of control (CON, including WT & HET) and MUT males to indicate mating activity. Each dot, the period between cohabitation and the detection of the first plug in the mated female; red line, median. (**b**) Percentage of fertile CON and MUT males. N = 15–17. (**c**) Litter size from fertile WT and MUT males. Error bar, standard deviation; N = 10–16. (**d**,**e**) Histology of 6 months old WT (**d**) and MUT (**e**) testes. Scale bar, 100 μm. (**f**) Sperm detection rate in reproductive tract of control females (N = 9–17) mated with WT (N = 4) or infertile MUT (N = 6) males. *P < 0.05. (**g**) Western blot in three uterine samples to verify a rabbit polyclonal anti-mouse LHFPL2 antibody. The main band detected was between 20 kD and 26 kD (predicated to be ~23.9 kD). (**h)** Immunohistochemistry detection of LHFPL2 in epithelium of PND7 vas deferens. (**i)** Immunohistochemistry in PND7 vas deferens using rabbit IgG as a negative control. Scale bar (**h,i**), 12.5 μm.

To speed up the breeding of MUT females, MUT males were mated with HET females but many did not sire offspring despite normal mating activity (Fig. 5a). Male fertility test by mating control and MUT males with control females revealed that ~70% of MUT males were infertile (Fig. 5b). The fertile MUT males produced normal litter sizes (Fig. 5c). Although the *Lhfpl2* MUT infertile males at 6-month old had slightly lower body weight and testis weight than their age-matched control males (see Supplementary Fig. S4a,b), their relative testis weight was comparable with that of the control (see Supplementary Fig. S4c) and there was no significant difference in the sperm counts from cauda epididymis (see Supplementary Fig. S4d) or the testis histology (Fig. 5d,e) between WT and sterile MUT males. However, none of the infertile males delivered sperms to the reproductive tract of their mated control females (Fig. 5f). These data demonstrated that the cause for MUT male infertility was not in the testis or epididymis, but beyond the cauda epididymis. Immunohistochemistry using an anti-LHFPL2 antibody (Fig. 5g) localized LHFPL2 in the epithelium of PND7 vas deferens (Fig. 5h,i).

### Distal vas deferens blockage caused by defective distal WD development in Lhfpl2 mutant males

**Figure 6:**
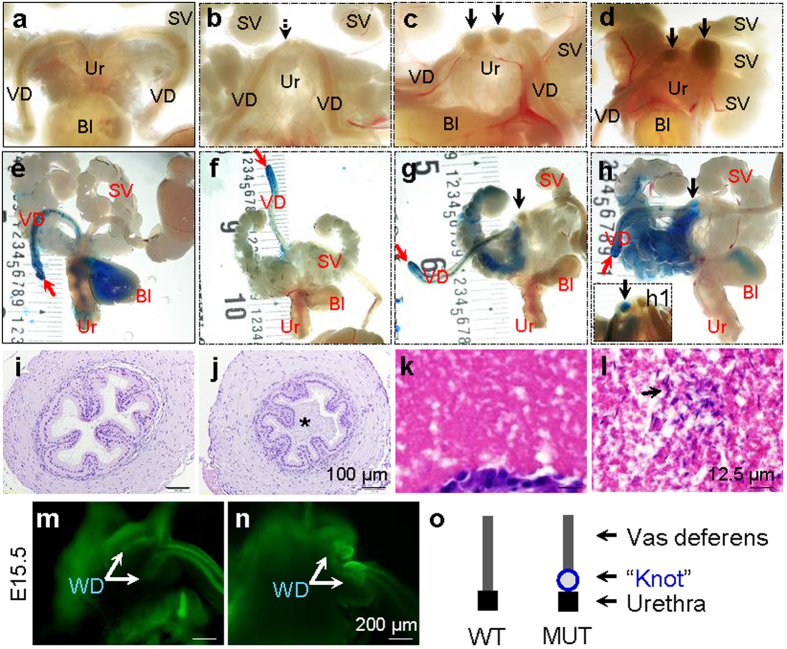
Male distal reproductive tract development. (**a–d)** Images of WT (**a**) and MUT (**b–d**) showing the junctions of vas deferens and urethra. (**e**) Injection of blue dye via WT vas deferens to indicate a passage to urethra. (**f–h1**). Injection of blue dye via MUT vas deferens to reveal various blockages to urethra. (**h1**) An insert in (**h**) to show blue dye traveling to a “knot” from another fertile MUT male. VD, vas deferens; Ur, urethra; SV, seminal vesicle; Bl, bladder; red arrow, blue dye injection site; dotted black arrow in (**b**), a less obvious “knot” at the junction of vas deferens and urethra; black arrow in (**c**,**d**) and (**g**-**h1**), visible “knot” at the junction of vas deferens and urethra. (**i**) Histology of adult WT vas deferens. (**j**) Histology of adult MUT vas deferens showing dense sperm in lumen. *Sperm; scale bar (**i,j**), 100 μm. (**k**) Histology of adult WT seminal vesicle showing absence of sperm. (**l)** Histology of adult MUT seminal vesicle showing sperm in the lumen. Black arrow, sperm (blue staining); scale bar (**k,l**), 12.5 μm. (**m**,**n**) PAX2 staining of WT (**m**) and MUT (**n**) in E15.5 male embryos. WD, Wolffian duct; scale bar, 200 μm. (**o**) Summary of phenotypes in male *Lhfpl2* MUT distal reproductive tract. Blue circle, a visible “knot” with distal vas deferens convolution; not shown, seminal vesicle abnormalities in 32% MUT males.

There was a smooth transition at the junctions of vas deferens and urethra with no visible “knots” in WT adult males (0/8) (Fig. 6a). In the 19 MUT adult male examined, 15 (15/19 = 79%) had clearly visible “knots” at the junctions of vas deferens and urethra (Fig. 6c,d), and the other four (4/19 = 21%) had small “knots” that can be identified with bright light under a dissecting scope (Fig. 6b). These “knots” indicated distal vas deferens convolution. In addition, 6 (6/19 = 32%) MUT males with visible “knots” also had abnormal seminal vesicles (e.g., 1–2 extra or tiny lobes of seminal vesicles) on one side or both sides (Fig. 6d and data not shown).

To reveal the passage from vas deferens to urethra, blue dye was injected through one vas deferens. The blue dye traveled through vas deferens to bladder and urethra but not the seminal vesicle in the WT (Fig. 6e). The blue dye traveling paths in the MUT males varied: no pass beyond vas deferens (Fig. 6f), traveling to seminal vesicle but not urethra or bladder (Fig. 6g), or traveling mainly to seminal vesicle and very limited to the bladder and urethra (Fig. 6h). The MUT males in the first (Fig. 6f) and second (Fig. 6g) scenarios were infertile while those in the third scenario (Fig. 6h) were fertile. Interestingly, it seemed that the blue dye could travel through the “knot” (Fig. 6h,h1) only in the fertile males. These data indicated that distal vas deferens convolution led to complete blockage in the infertile MUT males and incomplete blockage in the fertile MUT males. Histology of adult vas deferens showed that the MUT lumen (Fig. 6j) but not WT lumen (Fig. 6i) was filled with dense sperm. Sperm were detected in the MUT seminal vesicles (Fig. 6l) that the blue dye traveled to (Fig. 6g,h,h1) but not in WT seminal vesicles (Fig. 6k). PAX2 staining of E15.5 male embryos revealed that the two WT WD tips had individually entered the urogenital sinus (Fig. 6m), while the two MUT WD tips were enlarged and flattened and failed to enter the urogenital sinus (Fig. 6n). In addition, the tips of the MUT MDs were enlarged and apart while the WT MDs had merged and without enlargement (Fig. 6m,n). These data (Figs 5 and 6) demonstrated that the MUT WDs had no obvious defect in the triphasic process involving initiation, elongation, and differentiation; and that the defect in MUT WDs was at the WD tips that failed to merge with urogenital sinus, leading to convolution at the junctions of vas deferens and urethra (Fig. 6o) and abnormal seminal vesicles in some MUT males (Fig. 6d).

## Discussion

Vaginal opening has been used as an early indicator of pubertal onset in rodents. It can be regulated by estrogen signaling because estrogenic endocrine disruptors could promote vaginal opening^19^,^20^,^21^,^22^,^23^, while anti-estrogens could delay vaginal opening^29^,^30^ and estrogen signaling deficiency could lead to vaginal imperforation^31^ in rodents. Vaginal imperforation has been observed in several mouse models, such as β-catenin^C429S^ 32, double deficiency of Bak and Bax^33^, over-expression of Bcl2^34^, triple deficiency of Bid, Bim, and Puma^35^, triple deficiency of Tyro3, Axl, and Mer^36^, deficiency of p63^37^, Movo1^38^, Pax8^39^, Grb10^40^, Glypican-3^41^, Map3k1^42^, EphA1^43^, Vangl2^44^, or Lrp2/Megalin^31^. These genes have diverse functions, such as apoptosis, transcription, signal transduction, cell polarization, and endocytosis. Two mechanisms for vaginal imperforation in some of these models have been identified: lower vaginal atresia^32^ and prevention of apoptosis^34^. Estrogen-induced apoptosis has been proposed as a mechanism for vaginal opening during pubertal development^34^. Since exogenous E2 did not induce vaginal opening in Lrp2/Megalin-deficient mice^31^ similar as in this LHFPL2^G102E^ mouse strain with lower vaginal atresia, it is possible that vaginal atresia might be responsible for vaginal imperforation in Lrp2/Megalin-deficient mice.

Interestingly, β-catenin^C429S^ mice^32^ have several similar phenotypes with LHFPL2^G102E^ mice. For example, they both have normal oogenesis and spermatogenesis. They both have double-lumen upper vagina and no lower vagina, which occur in 91% β-catenin^C429S^ females and 100% LHFPL2^G102E^ females. β-catenin^C429S^ males are 100% infertile mainly due to abnormal seminal vesicles^32^. LHFPL2^G102E^ males are 70% infertile, but 100% of them have distal vas deferens convolution with complete or incomplete blockage, in addition to 32% of them having abnormal structure of seminal vesicles. These phenotypes in males point to defective development in the caudal WD, which differentiates into distal vas deferens and seminal vesicles^5^,^45^. Since LHFPL2^G102E^ WDs elongated but couldn’t merge with the urogenital sinus thus the WD tips became enlarged, and the remaining male reproductive tract had normal differentiation, it would suggest that the abnormal differentiation of distal vas deferens and seminal vesicle in the LHFPL2^G102E^ males resulted from failed merging with urogenital sinus.

It has been estimated that congenital vaginal atresia occurs in 1 of ~4000–5000 live female births in humans^46^. The mechanisms in human congenital vaginal atresia are unknown. Animal studies have provided clues for distal vaginal development. Lineage tracing study has demonstrated that the vagina is solely derived from MD in mice^47^. At birth, the vagina consists of “Müllerian vagina” (upper vagina) and “sinus vagina” (lower vagina). During postnatal development, the “Müllerian vagina” extends caudally following the migration of the “sinus vagina” towards the posterior end of the body. Although genetic fate mapping reveals that WD does not contribute cells to the MD^7^, there is caudal residual WD near MD^47^ that might guide the extension of MD to form the entire vagina^48^. The extension of MD depends on the cell proliferation of the caudal tip of the developing MD to deposit a cord of cells with mesoepithelial nature^7^.

Based on current knowledge, at least four mechanisms may explain vaginal atresia in the LHFPL2^G102E^ female and potentially in human. First, “sinus vagina” origin: “sinus vagina” fails to migrate towards the posterior end of the body so the “Müllerian vagina” could not extend to form the entire vagina. Second, MD origin: the caudal tip of the developing MD does not proliferate when it gets close to the urogenital sinus. However, the enlarged MD tips in both male and female E15.5 LHFPL2^G102E^ embryos would suggest that the MD tips could proliferate but could not extend to the urogenital sinus thus became enlarged. Third, WD origin: because the WD does not extend to the urogenital sinus in the LHFPL2^G102E^ embryos, the MD does not have a guide to extend. Fourth, defective communication: besides the three physical origins described above, it is possible that signaling molecules communicating between the urogenital sinus and MD might be altered. These mechanisms might also prevent the timely fusion of the upper vagina thus resulted in longitudinal upper vaginal septum in the LHFPL2^G102E^ females.

Male distal reproductive tract obstruction, such as distal vas deferens convolution and ejaculatory duct obstruction, is responsible for 1–5% of male infertility^49^. Although the molecular and cellular mechanisms for male distal reproductive tract development are also not well understood, mutations or deletion of CFTR (cystic fibrosis transmembrane conductance regulator), a membrane protein, are associated with a range of defects in vas deferens in mice^50^, pigs^51^, and humans^52^,^53^, indicating the critical role of CFTR in vas deferens development. In the LHFPL2^G102E^ males, the main anomaly in the reproductive tract is confined to the distal connection of the vas deferens, seminal vesicles, and urethra. The obvious defect is failed fusion of caudal WD with urogenital sinus, leading to the enlarged WD tips on E15.5 and distal vas deferens convolution shown as visible “knot” at the junction of vas deferens and urethra in adult.

According to a microarray database from GenitoUrinary Development Molecular Anatomy Project (GUDMAP), *Lhfpl2 *mRNA is expressed in the fetal male reproductive tract (WD) at all the time points checked between E12 and E18 (http://www.gudmap.org/gudmap/pages/mastertable_browse.html?gene =  Lhfpl2&geneId =  MGI%3A2145236&masterTableId =  4_7&cleartabs =  true). Since *Lhfpl2*/LHFPL2 is mainly detected in the epithelium of the postnatal and adult reproductive tracts, it is possible that *Lhfpl2*/LHFPL2 is also expressed in the epithelium of fetal reproductive tracts.

LHFP family members are tetraspan membrane proteins. It has been demonstrated that LHFPL5/TMHS is an integral component of the mechanotransduction machinery of cochlear hair cells regulating mechanotransduction in these cells and mutations in LHFPL5 cause hear loss^15^,^17^. Since a mutation in *Lhfpl2* (LHFPL2^G102E^) also leads to hear loss, it is possible that LHFPL2 might have a similar function as LHFPL5 in regulating mechanotransduction. Because mechanotransduction is a driving mechanism for organ morphogenesis^54^, LHFPL2 may play a role in mechanotransduction between the WDs and the urogenital sinus when the WD tips reach the urogenital sinus. The failed extension of WDs to the urogenital sinus in the LHFPL2^G102E^ embryos may simultaneously block the WD-guided extension of MDs to the urogenital sinus, eventually leads to defective distal reproductive tract development in both males and females.

Taken together, this study reveals that LHFPL2 is essential for distal reproductive tract development in both female and male mice and provides a novel genetic basis for distal reproductive tract development. This study also demonstrates that besides initiation, elongation and differentiation of MDs and WDs, an additional merging phase with urogenital sinus is essential for proper distal reproductive tract development. This LHFPL2^G102E^ mouse strain is a good model for studying mechanisms in distal reproductive tract development. It may also provide a genetic etiology for clinical syndromes, such as Park-Jones Syndrome associated with absent vagina and auditory anomaly^55^. It is interesting to investigate if LHFPL2 mutations are associated with longitudinal vaginal septum, vaginal agenesis, or obstructive azoospermia in humans.

## Methods

### Animals

*Lhfpl2* mutant mice (Stock No: 013716, LHFPL2^G102E^) in C57BL/6J background were purchased from the Jackson Laboratories (Bar Harbor, ME, USA) to establish a colony in Coverdell Vivarium at University of Georgia. All mice were housed in polypropylene cages with free access to food and water. The animal facility was maintained on a 12-hour light/dark cycle (0600 h to 1800 h) at 23 ± 1 °C with 30–50% relative humidity. All methods used in this study were approved by the University of Georgia Institutional Animal Care and Use Committee (IACUC) and conform to National Institutes of Health guidelines and public law.

### Genotyping

Genomic DNA was extracted using DirectPCR (Viagen, USA) according to manufacturer’s protocol. Two methods were used to determine the genotypes. One was PCR coupled with restriction digestion. A primer pair (Supplementary Table S1) flanking the point mutation on exon 3 amplified a 461 bp PCR product, which was subsequently digested by BglII (New England Biolabs, MA, USA) at 37 °C overnight. Undigested and digested products were run in 10% agrose gel. The digested wild type (WT) allele was expected to be 461 bp and the digested *Lhfpl2* mutant (MUT) allele was expected to be 189 bp and 272 bp. The other genotyping method was Custom Taqman SNP (single nucleotide polymorphism) genotyping assay (Life Technology, CA, USA). Taqman SNP genotyping primers were designed according to manufacturer’s instruction. VIC fluorescence represented WT allele; FAM fluorescence indicated MUT allele.

### LHFPL2 protein structure and secondary topology analyses

LHFPL2 protein blast was done using NCBI mouse protein database. LHFPL2 transmembrane prediction was performed using TMHMM Server v. 2.0 (http://www.cbs.dtu.dk/services/TMHMM/). LHFPL2 secondary topology was analyzed using SOSUI engine ver. 1.11 (http://bp.nuap.nagoya-u.ac.jp/sosui/sosui_submit.html).

### Generation of Lhfpl2 mutant transgenic mice

Transgenic mice in C57BL/6J background harboring wild type mouse *Lhfpl2* gene were generated at Cyagen Biosciences (CA, USA) using BAC ID#23-403I16. Three female and one male founders were produced. These female founders (WT/BAC+) were mated with pre-identified fertile *Lhfpl2* mutant males (MUT/BAC−) to generate *Lhfpl2* transgenic *Lhfpl2* heterozygous offspring (HET/BAC+). Female and male HET/BAC+ mice were mated to generate *Lhfpl2* transgenic *Lhfpl2* mutant offspring (MUT/BAC+). To determine the rescue effect of the transgene, the female MUT/BAC+ mice were monitored for vaginal opening, the male MUT/BAC+ mice were either tested for fertility or injected with blue dye via vas deferens to visualize the passage of the distal reproductive tract as described below.

### Visualization of reproductive tract

To visualize MUT vagina, the MUT female reproductive tract was dissected and Evans blue dye (0.05% in 1 × PBS) was injected via a blunt needle into both uterine horns and was accumulated in the two compartments of the upper vagina. To visualize the passage in male distal reproductive tract, the male reproductive system together with urethra were dissected and submerged into sesame oil in a petri dish. Under a dissecting microscope, Evans blue dye was injected into one vas deferens using a blunt needle. The distribution of the blue dye upon injection revealed whether or not there was a blockage in the male distal reproductive tract. Images were taken with a Nikon digital camera.

### 17β-estradiol (E2) treatment

Newly weaned WT and MUT females (3 weeks old) were i.p. injected with 100 μl sesame oil (vehicle) or 12.5 μg E2 (Sigma-Aldrich, USA) in vehicle for 3 consecutive days. Vaginal opening was monitored daily. All females were sacrificed the day after the third injection. Body weight, the weight of the female reproductive system (FRS, including ovary, oviduct, uterus, fluid, and vagina), the weight of the fluid in the female reproductive tract, which was determined by the difference between the FRS weight above and the FRS weight after the fluid was drained, and the weight of the uterine horns. Uterine tissues were fixed in formalin for histology. Relative weight was determined by dividing each weigh with body weight. N = 5–9.

### Collection of vaginas

WT and MUT females (N = 3) were sacrificed on postnatal day (PND) 3, 7, 14, 21, and 28. Vaginas were fixed in Formalin and examined for vaginal atresia. Longitudinal sections of PND28 vaginas and cross sections of PND7 upper vaginas were obtained.

### Perfusion

WT females at estrus stage, which was determined by vaginal smear^56^, WT and MUT females at 8 weeks old were perfused transcardially as previously described^57^. Cross sections of the uterus and upper vagina were obtained.

### Histology

Paraffin sections (5 μm) were deparaffinized, rehydrated, and stained with hematoxylin and eosin as previously described^19^.

### RT-PCR

The gene specific primers encompassing different exons were listed in Supplementary Table S1. Total RNAs from the following tissues (8 weeks old males and females, and 3 weeks old females), heart, spleen, liver, kidney, thymus, testis, brain, lung, intestine, vagina, ovary and uterus, were extracted using TRIzol (Invitrogen, Carlsbad, CA, USA). RT-PCR was done as previously described^58^,^59^. PCR products were visualized by electrophoresis in 10% agrose gel. *Hprt1* (hypoxanthine phosphoribosyltransferase 1) was used as a loading control.

### *In situ* hybridization

*In situ* hybridization was performed as previously described in frozen sections from female reproductive system^60^. Sense and antisense probes for *Lhfpl2* were synthesized from cDNA fragment amplified with a specific *Lhfpl2* primer pair (Table S1) and confirmed by sequencing (Genewiz, USA).

### Male mating activity, fertility, sperm delivery test, and sperm count

To determine the mating activity of the males (2 months old), control (WT/HET, N = 8) and MUT (N = 10) males were each housed with 3 virgin control (WT/HET, 2–4 months old) females, which were checked for a vaginal plug next morning. Plugging latency (the number of days between the start of cohabitation and the appearance of the first vaginal plug from each male) was recorded. To determine male fertility, each male (4 months old; control, N = 15; MUT, N = 17) was housed with 3 virgin control (WT/HET, 2–4 months old) females for 2 months to determine if it could sire offspring. The percentage of fertile males in each group was determined. Litter sizes were recorded from 16 litters sired by 6 WT males and 10 litters sired by 5 fertile MUT males. To examine sperm delivery, young control males (N = 4) and infertile MUT males (N = 6) were mated with control females. Upon identification of the first vaginal plug from a mated male, the female reproductive tract was flushed with 1 × PBS and the flushing was examined for the presence of sperm. Sperm counts were from cauda epididymis of 6 months old WT and infertile MUT males (N = 7), whose body weight and testis weight were also recorded.

### Western blot

Uterine samples (N = 3) from gestation day 3.5 WT mice were homogenized in RIPA buffer with protease inhibitor (1:100). Protein concentrations were measured by Bradford assay with Nanodrop. Samples (30 μg) were loaded into polyacrylamide gel. Separated proteins were transferred onto PVDF membrane and blocked with 5% non-fat milk for 1 hour at room temperature on an orbital shaker. After washed with the Tris-buffered saline containing 0.1% tween (TBST), the membrane was incubated in the primary rabbit polyclonal anti-mouse LHFPL2 antibody (1:200, PA5-25166, Thermo Fisher Scientific) over 2 nights at 4 °C in TBST containing 1% BSA (bovine serum albumin) on an orbital shaker. The membrane was then washed and incubated with the appropriate peroxidase-labeled secondary antibody (1:3000) diluted in 5% non-fat milk. The image was developed on the film after incubation with Pierce ECL western blotting substrate.

### Immunohistochemsitry

Immunohistochemistry was performed as previously described^61^. Briefly, PND7 vas deferens frozen sections (10 μm) were mounted, fixed, and subjected to antigen retrieval. Non-specific staining was blocked. Sections were then incubated with rabbit polyclonal anti-mouse LHFPL2 antibody (1:250, PA5-25166, Thermo Fisher Scientific) in blocking reagent at 4 °C for overnight, washed in 1 × PBS and incubated with biotinylated goat anti-rabbit secondary antibody (1:200 Santa Cruz Biotechnology, USA) for 30 min at room temperature. Sections were then incubated with ABComplex/HRP (Santa Cruz Biotechnology, USA) for 30 min at room temperature, washed, incubated with 3, 3′-diaminobenzidine tetrahydrochloride, counterstained with hematoxylin, and mounted for imaging. Negative control using serial sections was processed exactly the same except that the primary antibody was replaced with normal rabbit IgG.

### Whole-mount Immunofluorescence

Whole-mount immunofluorescence was performed mainly following a published protocol^62^. Briefly, embryonic day 15.5 (E15.5) WT and MUT urogenital system was isolated, fixed in 4% paraformaldehyde for overnight, washed in 1 × PBS with rotation, blocked with blocking solution (5% goat serum and 0.1% Triton X-100 in 1 × PBS) for 6 hours, and incubated with rabbit anti-PAX-2 (paired box gene 2) antibody (1:300, Cat No. 901001, BioLegend, San Diego, CA, USA) overnight. After washing (1% goat serum and 0.1% Triton X-100 in 1 × PBS) for 6 hours, the tissues were incubated with Alexa Fluor^®^488 (1:300, Invitrogen, Carlsbad, CA, USA) for 6 hours, washed and mounted on a slide for imaging. All the steps from fixation were performed at 4 °C. Images were taken under a fluorescent microscopy (Axio ScopeA1, Zeiss, USA).

### Statistical analyses

Two-tail, unequal variance Student’s t test was used to compare means and chi-square test was used to compare rates. The significant level was set at p < 0.05.

## Additional Information

**How to cite this article**: Zhao, F. *et al.* Novel function of LHFPL2 in female and male distal reproductive tract development. *Sci. Rep.* **6**, 23037; doi: 10.1038/srep23037 (2016).

## Supplementary Material

Supplementary Information

## References

[b1] Knobil E. & Neill J. D. The Physiology of reproduction. 2nd edn, (Raven Press, 1994).

[b2] Connell M., Owen C. & Segars J. Genetic Syndromes and Genes Involved in the Development of the Female Reproductive Tract: A Possible Role for Gene Therapy. J. Genet. Syndr. Gene Ther. 4 (2013). 10.4172/2157-7412.1000127 PMC4264624 25506511

[b3] Vohra S. & Morgentaler A. Congenital anomalies of the vas deferens, epididymis, and seminal vesicles. Urology 49, 313–321, 10.1016/S0090-4295(96)00433-5 (1997). 9123691

[b4] Kobayashi A. & Behringer R. R. Developmental genetics of the female reproductive tract in mammals. Nat. Rev. Genet. 4, 969–980, 10.1038/nrg1225 (2003). 14631357

[b5] Hannema S. E. & Hughes I. A. Regulation of Wolffian duct development. Horm. Res. 67, 142–151, 10.1159/000096644 (2007). 17077643

[b6] Murashima A., Xu B. & Hinton B. T. Understanding normal and abnormal development of the Wolffian/epididymal duct by using transgenic mice. Asian J Androl 17, 749–755, 10.4103/1008-682X.155540 (2015). 26112482 PMC4577584

[b7] Orvis G. D. & Behringer R. R. Cellular mechanisms of Mullerian duct formation in the mouse. Dev. Biol. 306, 493–504, 10.1016/j.ydbio.2007.03.027 (2007). 17467685 PMC2730733

[b8] Ma L. Endocrine disruptors in female reproductive tract development and carcinogenesis. Trends Endocrinol. Metab. 20, 357–363, 10.1016/j.tem.2009.03.009 (2009). 19709900 PMC2774851

[b9] Kurita T. Normal and abnormal epithelial differentiation in the female reproductive tract. Differentiation 82, 117–126, 10.1016/j.diff.2011.04.008 (2011). 21612855 PMC3178098

[b10] Petit M. M. *et al.* LHFP, a novel translocation partner gene of HMGIC in a lipoma, is a member of a new family of LHFP-like genes. Genomics 57, 438–441, 10.1006/geno.1999.5778 (1999). 10329012

[b11] Kalay E. *et al.* Mutations in the lipoma HMGIC fusion partner-like 5 (LHFPL5) gene cause autosomal recessive nonsyndromic hearing loss. Hum. Mutat. 27, 633–639, 10.1002/humu.20368 (2006). 16752389

[b12] Shabbir M. I. *et al.* Mutations of human TMHS cause recessively inherited non-syndromic hearing loss. Journal of medical genetics 43, 634–640, 10.1136/jmg.2005.039834 (2006). 16459341 PMC2564584

[b13] Longo-Guess C. M. *et al.* A missense mutation in the previously undescribed gene Tmhs underlies deafness in hurry-scurry (hscy) mice. Proc. Natl. Acad. Sci. USA 102, 7894–7899, 10.1073/pnas.0500760102 (2005). 15905332 PMC1142366

[b14] Longo-Guess C. M., Gagnon L. H., Fritzsch B. & Johnson K. R. Targeted knockout and lacZ reporter expression of the mouse Tmhs deafness gene and characterization of the hscy-2J mutation. Mammalian genome: official journal of the International Mammalian Genome Society 18, 646–656, 10.1007/s00335-007-9049-x (2007). 17876667 PMC2613174

[b15] Xiong W. *et al.* TMHS is an integral component of the mechanotransduction machinery of cochlear hair cells. Cell 151, 1283–1295, 10.1016/j.cell.2012.10.041 (2012). 23217710 PMC3522178

[b16] Cosgrove D. & Zallocchi M. Usher protein functions in hair cells and photoreceptors. Int. J. Biochem. Cell Biol. 46, 80–89, 10.1016/j.biocel.2013.11.001 (2014). 24239741 PMC3971483

[b17] Beurg M., Xiong W., Zhao B., Muller U. & Fettiplace R. Subunit determination of the conductance of hair-cell mechanotransducer channels. Proc. Natl. Acad. Sci. USA 112, 1589–1594, 10.1073/pnas.1420906112 (2015). 25550511 PMC4321290

[b18] Mansouri M. R. *et al.* Molecular genetic analysis of a *de novo* balanced translocation t(6;17)(p21.31;q11.2) associated with hypospadias and anorectal malformation. Hum. Genet. 119, 162–168, 10.1007/s00439-005-0122-9 (2006). 16395596

[b19] Zhao F. *et al.* Postweaning exposure to dietary zearalenone, a mycotoxin, promotes premature onset of puberty and disrupts early pregnancy events in female mice. Toxicol. Sci. 132, 431–442, 10.1093/toxsci/kfs343 (2013). 23291560 PMC3595522

[b20] Zhao F. *et al.* Multigenerational exposure to dietary zearalenone (ZEA), an estrogenic mycotoxin, affects puberty and reproduction in female mice. Reprod. Toxicol. 47C, 81–88, 10.1016/j.reprotox.2014.06.005 (2014). 24972337 PMC4137769

[b21] Zhao F. *et al.* Timing and recovery of postweaning exposure to diethylstilbestrol on early pregnancy in CD-1 mice. Reprod. Toxicol. 49C, 48–54, 10.1016/j.reprotox.2014.07.072 (2014). 25062584 PMC4303565

[b22] Li R., Zhao F., Diao H., Xiao S. & Ye X. Postweaning dietary genistein exposure advances puberty without significantly affecting early pregnancy in C57BL/6J female mice. Reprod. Toxicol. 44, 85–92, 10.1016/j.reprotox.2013.12.003 (2014). 24365114 PMC4004695

[b23] Li R., El Zowalaty A. E., Chen W., Dudley E. A. & Ye X. Segregated responses of mammary gland development and vaginal opening to prepubertal genistein exposure in Bscl2(-/-) female mice with lipodystrophy. Reprod. Toxicol. 54, 76–83, 10.1016/j.reprotox.2014.10.023 (2015). 25462787 PMC4420718

[b24] Rollerova E., Wsolova L. & Urbancikova M. Neonatal exposure to herbicide acetochlor alters pubertal development in female wistar rats. Toxicol. Mech. Methods 21, 406–417, 10.3109/15376516.2010.551554 (2011). 21320039

[b25] Ojeda S. R., Advis J. P. & Andrews W. W. Neuroendocrine control of the onset of puberty in the rat. Federation proceedings 39, 2365–2371 (1980). 6989642

[b26] Stoker T. E. *et al.* Assessment of DE-71, a commercial polybrominated diphenyl ether (PBDE) mixture, in the EDSP male and female pubertal protocols. Toxicological sciences: an official journal of the Society of Toxicology 78, 144–155, 10.1093/toxsci/kfh029 (2004). 14999130

[b27] Stoker T. E., Gibson E. K. & Zorrilla L. M. Triclosan exposure modulates estrogen-dependent responses in the female wistar rat. Toxicol. Sci. 117, 45–53, 10.1093/toxsci/kfq180 (2010). 20562219

[b28] Walters L. M., Rourke A. W. & Eroschenko V. P. Purified methoxychlor stimulates the reproductive tract in immature female mice. Reprod. Toxicol. 7, 599–606 (1993). 8118110 10.1016/0890-6238(93)90036-7

[b29] Chadwick R. W., Cooper R. L., Chang J., Rehnberg G. L. & McElroy W. K. Possible antiestrogenic activity of lindane in female rats. J. Biochem. Toxicol. 3, 147–158 (1988). 2462049 10.1002/jbt.2570030303

[b30] Ashby J., Tinwell H., Stevens J., Pastoor T. & Breckenridge C. B. The effects of atrazine on the sexual maturation of female rats. Regul. Toxicol. Pharmacol. 35, 468–473 (2002). 12202059 10.1006/rtph.2002.1571

[b31] Hammes A. *et al.* Role of endocytosis in cellular uptake of sex steroids. Cell 122, 751–762, 10.1016/j.cell.2005.06.032 (2005). 16143106

[b32] Murata T. *et al.* beta-CateninC429S mice exhibit sterility consequent to spatiotemporally sustained Wnt signalling in the internal genitalia. Sci. Rep. 4, 6959, 10.1038/srep06959 (2014). 25376241 PMC4223658

[b33] Lindsten T. *et al.* The combined functions of proapoptotic Bcl-2 family members bak and bax are essential for normal development of multiple tissues. Mol. Cell 6, 1389–1399 (2000). 11163212 10.1016/s1097-2765(00)00136-2 PMC3057227

[b34] Rodriguez I., Araki K., Khatib K., Martinou J. C. & Vassalli P. Mouse vaginal opening is an apoptosis-dependent process which can be prevented by the overexpression of Bcl2. Dev. Biol. 184, 115–121, 10.1006/dbio.1997.8522 (1997). 9142988

[b35] Ren D. *et al.* BID, BIM, and PUMA are essential for activation of the BAX- and BAK-dependent cell death program. Science 330, 1390–1393, 10.1126/science.1190217 (2010). 21127253 PMC3163443

[b36] Wu H., Tang H., Chen Y., Wang H. & Han D. High incidence of distal vaginal atresia in mice lacking Tyro3 RTK subfamily. Mol. Reprod. Dev. 75, 1775–1782, 10.1002/mrd.20917 (2008). 18393392

[b37] Ince T. A. *et al.* p63 Coordinates anogenital modeling and epithelial cell differentiation in the developing female urogenital tract. Am. J. Pathol. 161, 1111–1117, 10.1016/S0002-9440(10)64387-8 (2002). 12368184 PMC1867285

[b38] Dai X. *et al.* The ovo gene required for cuticle formation and oogenesis in flies is involved in hair formation and spermatogenesis in mice. Genes Dev. 12, 3452–3463 (1998). 9808631 10.1101/gad.12.21.3452 PMC317232

[b39] Mittag J., Winterhager E., Bauer K. & Grummer R. Congenital hypothyroid female pax8-deficient mice are infertile despite thyroid hormone replacement therapy. Endocrinology 148, 719–725, 10.1210/en.2006-1054 (2007). 17082261

[b40] Holt L. J. *et al.* Grb10 regulates the development of fiber number in skeletal muscle. FASEB J. 26, 3658–3669, 10.1096/fj.11-199349 (2012). 22623587

[b41] Cano-Gauci D. F. *et al.* Glypican-3-deficient mice exhibit developmental overgrowth and some of the abnormalities typical of Simpson-Golabi-Behmel syndrome. J. Cell Biol. 146, 255–264 (1999). 10402475 10.1083/jcb.146.1.255 PMC2199732

[b42] Warr N. *et al.* Minor abnormalities of testis development in mice lacking the gene encoding the MAPK signalling component, MAP3K1. PLoS One 6, e19572, 10.1371/journal.pone.0019572 (2011). 21559298 PMC3086927

[b43] Duffy S. L. *et al.* Generation and characterization of EphA1 receptor tyrosine kinase reporter knockout mice. Genesis 46, 553–561, 10.1002/dvg.20434 (2008). 18802966

[b44] Vandenberg A. L. & Sassoon D. A. Non-canonical Wnt signaling regulates cell polarity in female reproductive tract development via van gogh-like 2. Development 136, 1559–1570, 10.1242/dev.034066 (2009). 19363157 PMC2674261

[b45] Staack A., Donjacour A. A., Brody J., Cunha G. R. & Carroll P. Mouse urogenital development: a practical approach. Differentiation 71, 402–413, 10.1046/j.1432-0436.2003.7107004.x (2003). 12969333

[b46] Zafar M. *et al.* Use of amnion in vaginoplasty for vaginal atresia. Journal of the College of Physicians and Surgeons–Pakistan: JCPSP 17, 107–109, 02.2007/JCPSP.107109 (2007). 17288860

[b47] Kurita T. Developmental origin of vaginal epithelium. Differentiation 80, 99–105, 10.1016/j.diff.2010.06.007 (2010). 20638775 PMC2943051

[b48] Drews U. Helper function of the Wolffian ducts and role of androgens in the development of the vagina. Sex. Dev. 1, 100–110, 10.1159/000100031 (2007). 18391520

[b49] Cocuzza M., Alvarenga C. & Pagani R. The epidemiology and etiology of azoospermia. Clinics 68 Suppl 1, 15–26 (2013). 23503951 10.6061/clinics/2013(Sup01)03 PMC3583160

[b50] Reynaert I. *et al.* Morphological changes in the vas deferens and expression of the cystic fibrosis transmembrane conductance regulator (CFTR) in control, deltaF508 and knock-out CFTR mice during postnatal life. Mol. Reprod. Dev. 55, 125–135, 10.1002/(SICI)1098-2795(200002)55:2<125::AID-MRD1>3.0.CO;2-Q(2000 ). 10618651

[b51] Pierucci-Alves F. *et al.* Swine models of cystic fibrosis reveal male reproductive tract phenotype at birth. Biology of reproduction 85, 442–451, 10.1095/biolreprod.111.090860 (2011). 21593481 PMC3159534

[b52] Yang X. *et al.* Novel mutations and polymorphisms in the CFTR gene associated with three subtypes of congenital absence of vas deferens. Fertil. Steril. 104, 1268–1275 e1262, 10.1016/j.fertnstert.2015.07.1143 (2015). 26277102

[b53] Yu J., Chen Z., Ni Y. & Li Z. CFTR mutations in men with congenital bilateral absence of the vas deferens (CBAVD): a systemic review and meta-analysis. Hum. Reprod. 27, 25–35, 10.1093/humrep/der377 (2012). 22081250

[b54] Mammoto T. & Ingber D. E. Mechanical control of tissue and organ development. Development 137, 1407–1420, 10.1242/dev.024166 (2010). 20388652 PMC2853843

[b55] Park I. J., Jones H. W., Nager G. T., Chen S. C. & Hussels I. E. A new syndrome in two unrelated females: Klippel-Feil deformity, conductive deafness and absent vagina. Birth Defects Orig Artic Ser 7, 311–317 (1971). 5173188

[b56] Byers S. L., Wiles M. V., Dunn S. L. & Taft R. A. Mouse estrous cycle identification tool and images. PLoS One 7, e35538, 10.1371/journal.pone.0035538 (2012). 22514749 PMC3325956

[b57] Li R. *et al.* Olfactomedin 1 Deficiency Leads to Defective Olfaction and Impaired Female Fertility. Endocrinology 156, 3344–3357, 10.1210/en.2015-1389 (2015). 26107991 PMC4541623

[b58] El Zowalaty A. E. *et al.* Seipin deficiency increases chromocenter fragmentation and disrupts acrosome formation leading to male infertility. Cell Death Dis. 6, e1817, 10.1038/cddis.2015.188 (2015). 26181198 PMC4650735

[b59] Xiao S. *et al.* Differential gene expression profiling of mouse uterine luminal epithelium during periimplantation. Reprod. Sci. 21, 351–362, 10.1177/1933719113497287 (2014). 23885106 PMC3936419

[b60] Diao H., Xiao S., Zhao F. & Ye X. Uterine luminal epithelium-specific proline-rich acidic protein 1 (PRAP1) as a marker for successful embryo implantation. Fertil. Steril. 94, 2808–2811 e2801, S0015-0282(10)00987-8 (2010). 20674898 10.1016/j.fertnstert.2010.06.034

[b61] Diao H., Paria B. C., Xiao S. & Ye X. Temporal expression pattern of progesterone receptor in the uterine luminal epithelium suggests its requirement during early events of implantation. Fertil. Steril. 95, 2087–2093, S0015-0282(11)00239-1 (2011). 21371703 10.1016/j.fertnstert.2011.01.160 PMC3080439

[b62] Nicol B. & Yao H. H. Gonadal Identity in the Absence of Pro-Testis Factor SOX9 and Pro-Ovary Factor Beta-Catenin in Mice. Biol Reprod 93, 35, 10.1095/biolreprod.115.131276 (2015). 26108792 PMC4706297

