# Supplementary material for: Novel function of LHFPL2 in female and male distal reproductive tract development
Source: Sci Rep. 2016 Mar 11;6:23037. doi: 10.1038/srep23037 (PMC4786858; doi:10.1038/srep23037)
Supplement: Supplementary Information [file srep23037-s1.pdf]

Novel function of LHFPL2 in female and male distal reproductive tract development

Fei Zhao [1, 2, 3], Jun Zhou [1, 2], Rong Li [1, 2], Elizabeth A. Dudley [1,2], Xiaoqin Ye [1, 2, *]

[1] Department of Physiology and Pharmacology, College of Veterinary Medicine; [2] Interdisciplinary Toxicology Program, University of Georgia, Athens, GA 30602, USA; [3] Current address: Reproductive Developmental Biology Group, National Institute of Environmental Health Sciences (NIEHS/NIH), 111 TW Alexander Drive, Research Triangle Park, NC 27709, USA.

* Corresponding author: Xiaoqin Ye, M.D., Ph.D., 501 DW Brooks Dr., Department of Physiology and Pharmacology, College of Veterinary Medicine; Interdisciplinary Toxicology Program, University of Georgia, Athens, GA 30602, USA (Tel: 1-706-542-6745; Fax: 1-706-542-3015; E-mail: ye@uga.edu).

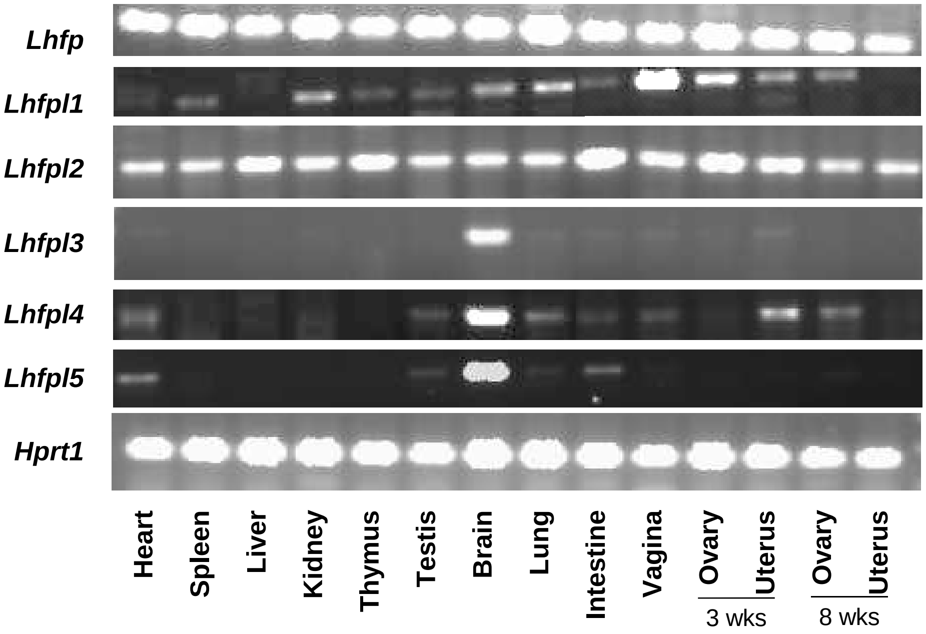

Zhao F et al.
Supplementary Figure S1

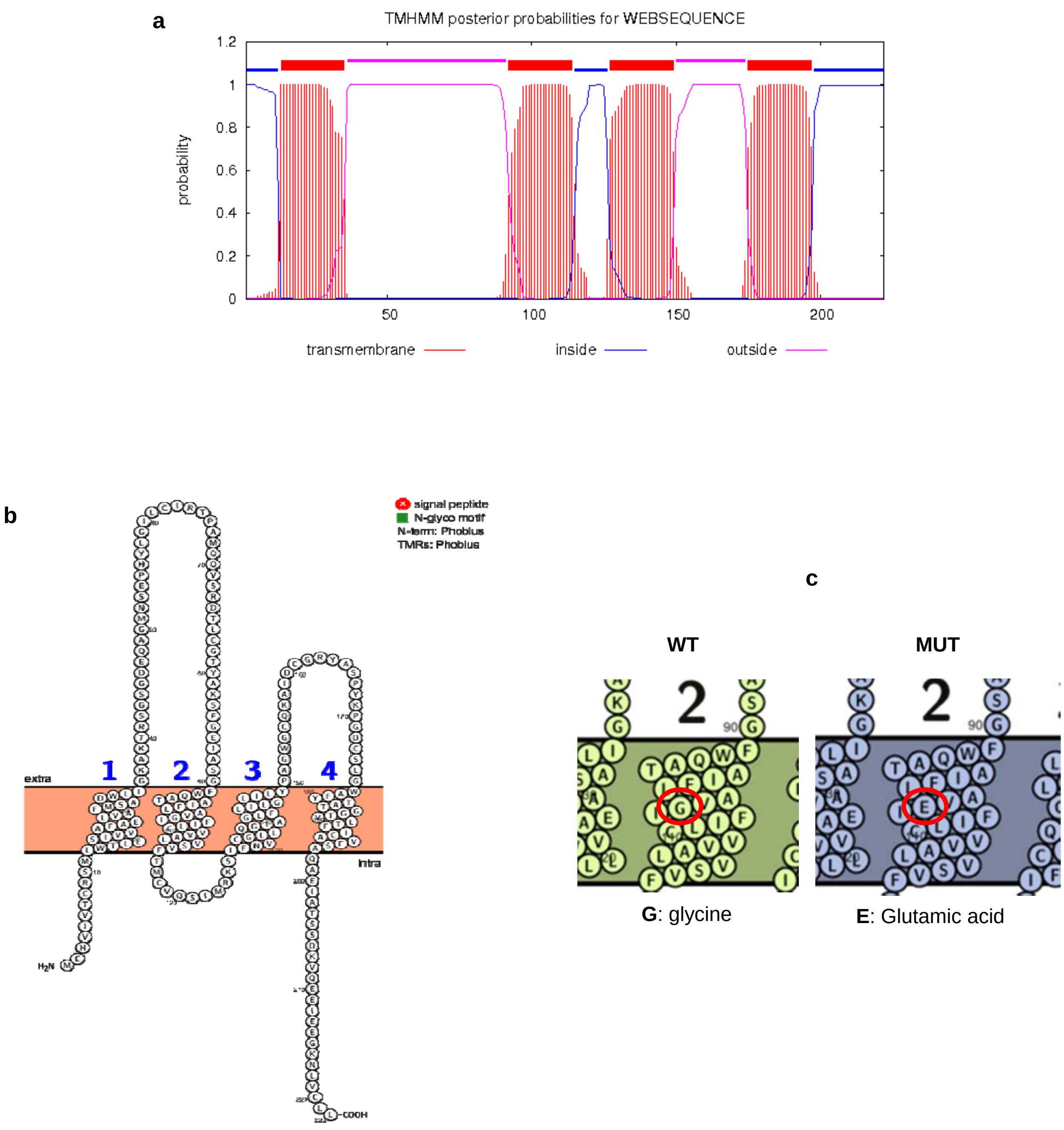

Zhao F et al.
Supplementary Figure S2

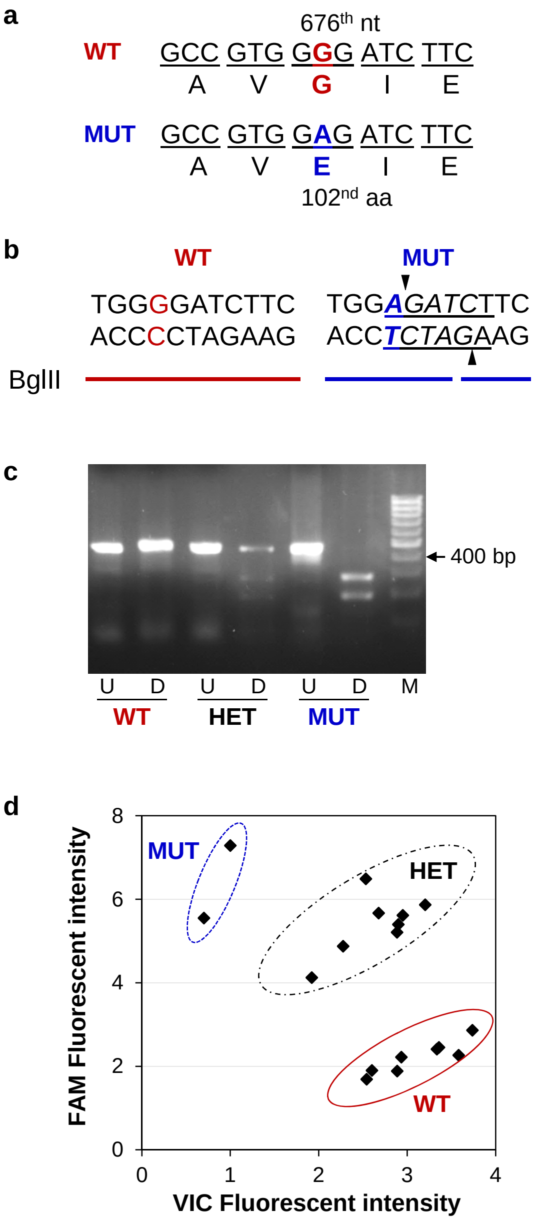

Zhao F et al.
Supplementary Figure S3

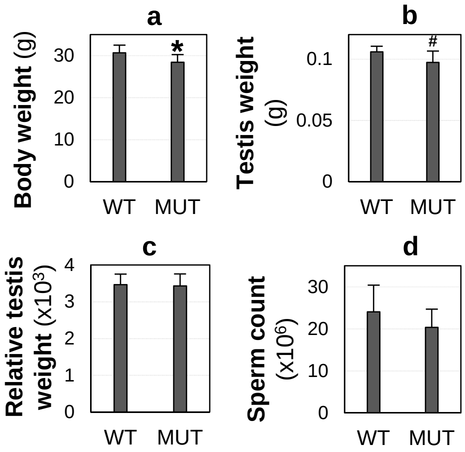

Zhao F et al.
Supplementary Figure S4

Supplementary Figure Legends

Supplementary Figure S1. Expression of *Lhfp* gene family members in wild type 8 weeks old mouse tissues and 3 weeks old ovary and uterus by RT-PCR. *Hprt1*, hypoxanthine phosphoribosyltransferase 1, a house-keeping gene.

Supplementary Figure S2. Predicated LHFPL2 structure. a. Transmembrane prediction indicating LHFPL2 as a tetra-transmembrane protein. b. Secondary topology predication of LHFPL2. c. Predicated transmembrane domain 2 showing a point mutation in amino acid 102 (red circle) in the mutant mice.

Supplementary Figure S3. Genotyping of *Lhflp2* mutation with restriction digestion of PCR product and custom Taqman SNP genotyping assay. WT, wild type; HET, heterozygote with one wild type allele and one mutant allele; MUT, mutant. a. The point mutation in full length *Lhfpl2* cDNA 676th nucleotide from G to A leading to the 102nd animo acid from glycine (G) to glutamic acid (E). b. A BglII restriction site from the point mutation and predicated DNA products from BglII digestion of PCR product covering the point mutation. c. Agarose gel image of the undigested (U) and BglII digested (D) PCR product covering the point mutation. WT had a single band; HET had one band from the WT allele and two smaller ones from the MUT allele; MUT had two smaller bands. M, molecular marker. d. A representative set of post-reading from custom Taqman SNP genotyping assay. X-axis, intensity of VIC fluorescence, representing WT allele; Y-axis, intensity of FAM fluorescence, representing MUT allele.

Supplementary Figure S4. A few parameters from 6 months old WT and infertile MUT males. a. Body weight. * P<0.05. b. Absolute testis weight. # P=0.0555. c. Relative testis weight. d. Sperm count from cauda epididymis. Error bar, standard deviation; N=7.

Supplementary Table S1. List of primers used in RT-PCR, point mutation confirmation, and *in situ* hybridization.

Primers	**Sequence**	Product size (bp)	Accession Number/Usage
mLhfp e2F1	TTCATGCCATACTGGCTCT	397	NM_175386.3/<br>RT-PCR
mLhfp e3R1	CAAACTGGCCAGAGATGTAG		
mLhfpl1 e2F2	TGGCCAGTTCTACCAGTTAC	393	NM_178358.3/<br>RT-PCR
mLhfpl1 e3R2	CGCATGTTTGCATTACCTC		
mLhfpl2 e3F1	AAGAGCTTCGGGGAGATAG	388	NM_172589.2/<br>RT-PCR<br>In situ hybridization
mLhfpl2 e4R1	CTTCCTGGACTTTGTCACTG		
mLhfpl3 e1F1	TAGCTTCACGGACTTCTCC	400	NM_029990.1<br>NM_001081231.2/<br>RT-PCR
mLhfpl3 e2R1	TTCTGCTTTCAGTTCCTCTG		
mLhfpl4 e2F2	ATCATCAACGTGGTGGTCT	399	NM_177763.3/<br>RT-PCR
mLhfpl4 e3R2	GGAGTACTTCCCTGTCTTGG		
mLhfpl5 e1F2	ACACCAACTATGTGCGAAAC	395	NM_026571.2/<br>RT-PCR
mLhfpl5 e2R2	TAGACATCCGATCATTAGGC		
conF: Lhfpl2 point mutation confirmation F	CTCTGGACCCTCCTGAGTAT	461	Digestion genotyping
conR: Lhfpl2 point mutation confirmation R	CTTCAACAGATGCAAGCAGT		
mHprt1 e3F1	GCTGACCTGCTGGATTACAT	172	NM_013556<br>NM_000194/<br>RT-PCR
mHprt1 e4/5R1	CAATCAAGACATTCTTTCCAGT		
